# A 21-Day Individual Rehabilitation Exercise Training Program Changes Irisin, Chemerin, and BDNF Levels in Patients after Hip or Knee Replacement Surgery

**DOI:** 10.3390/jcm12154881

**Published:** 2023-07-25

**Authors:** Bronisława Skrzep-Poloczek, Maciej Idzik, Katarzyna Michalczyk, Elżbieta Chełmecka, Michał Kukla, Jolanta Zalejska-Fiolka, Jakub Poloczek, Bartosz Bogielski, Jerzy Jochem, Damian Nowak, Dominika Stygar

**Affiliations:** 1 Department of Physiology, Faculty of Medical Sciences in Zabrze, Medical University of Silesia, Jordana 19 Street, 41-808 Zabrze, Poland; bskrzep-poloczek@sum.edu.pl (B.S.-P.); bartosz.bogielski@sum.edu.pl (B.B.);; 2 Independent Public Health Care, Opole Cancer Center Prof. Tadeusz Koszarowski, Katowicka 45-061 Street, 46-020 Opole, Poland; macidzik@tlen.pl; 3 Department of Statistics, Department of Instrumental Analysis, Faculty of Pharmaceutical Sciences in Sosnowiec, Medical University of Silesia, Ostrogórska 31 Street, 41-200 Sosnowiec, Poland; echelmecka@sum.edu.pl; 4 Department of Internal Medicine and Geriatrics, Jagiellonian University Medical College in Cracow, Jakubowskiego 28 Street, 31-501 Cracow, Poland; michal.kukla@uj.edu.pl; 5 Department of Endoscopy, University Hospital in Cracow, Jakubowskiego 28 Street, 31-501 Cracow, Poland; 6 Department of Biochemistry, Faculty of Medical Sciences in Zabrze, Medical University of Silesia, Jordana 19 Street, 41-808 Zabrze, Poland; jzalejskafiolka@sum.edu.pl; 7 Department of Rehabilitation, 3rd Specialist Hospital in Rybnik, Energetyków 46 Street, 44-200 Rybnik, Poland; 8 SLU University Animal Hospital, Swedish University of Agricultural Sciences, SE-750 07 Uppsala, Sweden

**Keywords:** brain-derived neurotrophic factor (BDNF), chemerin, hip or knee replacement surgery, irisin, individual rehabilitation exercise training, osteoarthritis

## Abstract

Osteoarthritis (OA) is the most frequent worldwide cause of adult population disabilities. The study evaluated the effects of a 21-day individual rehabilitation exercise training program focused on improving patients’ functional capacity. The study analyzed the changes in irisin, chemerin, and BDNF serum levels in 36 OA patients subjected to an individually-adjusted rehabilitation program 90 days after surgical hip or knee replacement. The changes in irisin, chemerin, and BDNF serum levels were measured using enzyme-linked immunosorbent assay (ELISA) kits. A 21-day individual rehabilitation exercise training program significantly increased irisin and BDNF, and decreased chemerin serum levels. The presented study indicates that individually-adjusted exercise training is an important modulator influencing serum levels of anti- and pro-inflammatory factors, leading to positive clinical outcomes in osteoarthritis therapy. Selected factors are considered potential markers of various pathophysiological conditions. The presented study brings new details to the discussion.

## 1. Introduction

Osteoarthritis (OA) is the most prevalent degenerative joint disease affecting the adult population. It is also one of the most important causes of adult disabilities worldwide. Osteoarthritis prevalence is associated with different factors, with metabolic syndrome being one of them [1]. Metabolic syndrome in OA patients influences biomechanics, dysregulates chondrocyte metabolism, and interplays between metabolic regulation and immune response, leading to further clinical complications [2].

Irisin is one of the recently discovered myokines identified as a marker of muscle weakness and atrophy [3]. It is mainly expressed and secreted by skeletal muscles as a product of the fibronectin type III domain containing 5 (FNDC5) cleavage [4]. Irisin levels depend on PPAR-γ (peroxisome proliferator-activated receptor gamma) coactivator-1-α (PGC1α), which is expressed after physical exertion. Increased PGC1α levels upregulate the expression of FNDC5, from which irisin is eventually derived [5,6]. Therefore, post-exercise irisin production exerts positive effects on the metabolism, and may play a beneficial role in treatment of obesity and obesity-related diseases, type 2 diabetes mellitus (T2DM), or non-alcoholic fatty liver disease (NAFLD) [6], especially since decreased irisin levels accompany obesity, type 2 T2D, and other diseases like chronic renal failure and prolonged hypothyroidism [7]. Some studies show that irisin is also produced by adipose tissue, and acts as adipokine [5,6,7]. As irisin levels decrease with age, irisin seems to be associated with a wide range of aging-related diseases [8,9,10,11].

Chemerin is an adipokine secreted by adipose, endothelial, synovial cells, and chondrocytes, and might show chemotherapeutic activity through the chemerin receptor 23 (Chem23) and increased TNF, IL1-β, IL-6, MMP-1, and MMP-8 expression [12]. It was reported that chemerin is associated with obesity, disease severity, inflammation, and cartilage destruction in patients with knee OA. The study showed that it was linked to obesity, BMI, joint inflammation, and cartilage degradation, independent of mechanical factors [13].

Brain-derived neurotrophic factor (BDNF) is a protein found in the brain and, peripherally, in the blood [14]. Peripherally, it is expressed by skeletal [15], adipose [16], and endothelial cells [17], and stored in a form bound to platelets in the blood, liver, and spleen [18]. As a highly conserved neurotrophic protein, it regulates synapses, affecting various brain regions structurally and functionally. It promotes neuron survival, neurite growth (the process by which developing neurons form new processes), and synapse formation [19,20,21], ensuring neuroplasticity, learning, and memory. It also plays a role in the hypothalamic signaling pathway: it controls body weight, decreases food intake, and lowers blood glucose levels, thus controlling energy homeostasis [22,23]. About 70–80% of circulating plasma BDNF originates from the brain, both during exercise and recovery [22].

Each of the above-mentioned factors seems to be related to physical activity, age-related diseases, or energy homeostasis. Different types of physical activity increase the release of myokines, including irisin, which, aside from other functions, stimulates the metabolism of energy-related signaling and memory formation-related signaling like BDNF [24].

New therapies of degenerative disorders, like rheumatoid arthritis (RA), include nanomedical management [25]. However, we hypothesized that physical therapy, which usually provides good results for OA patients [26], in the form of moderate, controlled exercise training after hip or knee replacement in OA patients with chronic pain would improve the profile of irisin, chemerin, and brain-derived neurotrophic factor (BDNF). The study aimed to determine the efficacy of individual rehabilitation exercise training in improving the functional capacity of patients who underwent hip or knee replacement surgery, and the accompanying changes in irisin, chemerin, and BDNF levels after a 21-day individually-adjusted exercise rehabilitation program.

## 2. Materials and Methods

### 2.1. Ethical Statement and Permissions

The study followed the Declaration of Helsinki guidelines and was approved by the Ethics Committee of the Medical University of Silesia in Katowice (N° KNW/002/KB1/106/17; 3 October 2017). Every participant of the study received the study protocol description, was informed about its benefits and possible risks, and returned the written informed consent before the study started.

### 2.2. Study Group

The participants were recruited over 2017–2018 from the outpatient clinic and the Department of Rehabilitation at the 3rd Specialist Hospital in Rybnik. The clinical interview carried out during the recruitment process excluded patients with inflammatory disorders, infections, renal or hepatic insufficiency, active coronary artery disease, diabetes, heart failure, hormonal replacement therapy, or supplementation with antioxidants taken up to 3 months before the study. Eventually, 41 patients after total hip (*n* = 29) or knee (*n* = 12) replacement, aged 61.0 ± 8.1 years, 22 men and 19 women, were included in the study (Table 1). On the initial rehabilitation day, the patients were 89.6 ± 9.7 days after the joint replacement surgery. On the first day they arrived at the outpatient clinic, the resting electrocardiogram (ECG) and blood pressure measurement were recorded, and the body mass and height measurements were taken. Later on, five patients were excluded from the irisin, chemerin, and BDNF analyses, due to health conditions that occurred during the study.

### 2.3. Individual Rehabilitation Exercise Training

All patients underwent a 21-day individual rehabilitation exercise training program. The daily rehabilitation sessions started between 8:00 and 8:45 a.m. The individual rehabilitation exercise training mainly consisted of physiotherapy, and living activities training focused on improving the patients’ walking functionality: lengthening stride, increasing pace, walking backward and on uneven surfaces, climbing stairs, and Nordic walking. Additionally, the rehabilitation program included patients’ nutritional education. The individual rehabilitation sessions comprised 30–45 min of aerobic walking, 20–30 min of strength training, 30–45 min of rotor/bicycle training, and a 15 min cool-down phase. The patients were instructed to continue the learned activities at home, to keep their physical fitness and biochemical parameters at the beneficial level [27]. The choice of exercises (different strength and balance exercises) and training modalities (number and sets of repetitions as well as the duration of resting time) were individually adjusted to each patient, then monitored in the rehabilitation by the responsible physiotherapist.

### 2.4. Samples Collection

Blood samples were collected before the initial and after the final (after the patient’s HR returned to the resting value) rehabilitation sessions. A blood sample (5 mL) from the ulnar vein was collected to the standard blood tubes with a clot activator (S-Monovette, SARSTEDT). The samples for serum analysis were centrifuged at 4000 rpm for 10 min at 4 °C and then subsequently frozen and stored at −80 °C until further analyses could be performed.

### 2.5. Irisin, Chemerin, and Brain-Derived Neurotrophic Factor (BDNF) Assessment

Irisin and chemerin concentrations were assessed using an enzyme-linked immunoabsorbent assay (ELISA) kit (cat. no. RAG018R and RD191136200R, respectively, BioVendor, Brno, Czech Republic). BDNF concentration was assessed using an enzyme-linked immunoabsorbent assay (ELISA) kit (cat. no. SEA011Hu, Cloud Clone Corp., Katy, TX, USA). The serum samples and all reagents were prepared and processed as per the manufacturers’ guidelines. The color change in the samples was measured spectrophotometrically using the microplate reader (BioTek Synergy HTX Multimode Reader, BioTek^®^ Instruments, Inc., Winooski, VT, USA). The results were calculated as per the manufacturers’ guidelines, using dedicated Gen 5 Microplate Data Collection and Analysis Software ver. 3.14.03 (BioTek^®^ Instruments, Inc., Winooski, VT, USA). The results for irisin were read against an 8-point calibration curve ranging from 0.001–5 μg irisin/mL. Intra-assay precision was CV < 7%, inter-assay precision was CV < 10%, and the lower limit of detection was 1 ng irisin/mL. The results for chemerin were read against a 6-point calibration curve ranging from 0.25–8 ng chemerin/mL. Intra-assay precision was CV = 6%, inter-assay precision was CV = 7.6%, and the lower limit of detection was 1 ng irisn/mL.

The results for BDNF were read against a 7-point calibration curve ranging from 0.156–10 ng BDNF/mL. Intra-assay precision was CV < 10%, inter-assay precision was CV < 12%, and the lower limit of detection was 0.061 ng BDNF/mL.

### 2.6. Statistical Analysis

The analysis was performed, and graphs were created, using Statistica ver. 13.0 (TIBCO Software Inc., Palo Alto, CA, USA). Data distribution was assessed using the Shapiro–Wilk test and quantile–quantile plots. The mean values with standard deviation (SD) were calculated for normally distributed data, and the median and lower-upper quartile (Me (Q1–Q3)) for non-normal distributed data. The non-normal distributed data were log-transformed. The *t*-test for related samples was used to compare the parameters before and after rehabilitation. All tests were two-tailed. Statistical significance was set at *p* < 0.05.

## 3. Results

**Figure 1:**
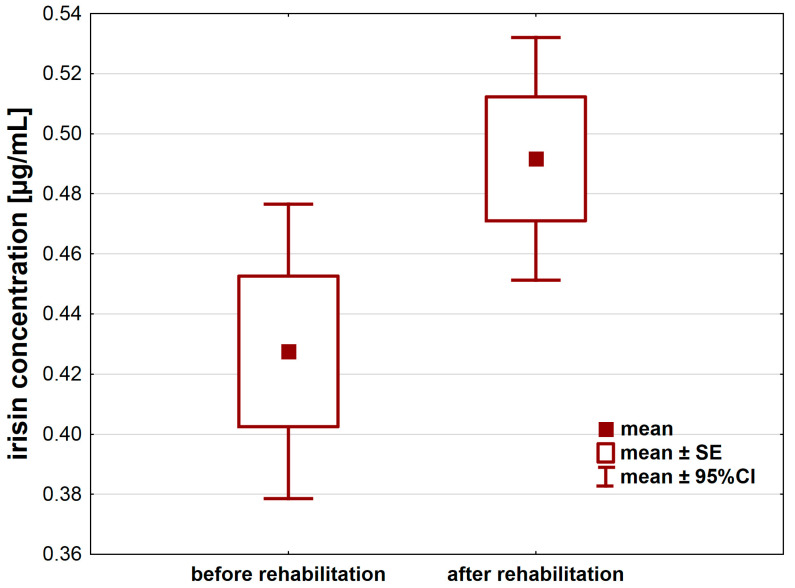
Irisin concentration [µg/mL] in the serum of patients after hip or knee replacement surgery enrolled in a 21-day individual rehabilitation exercise training program.

A 21-day individual rehabilitation exercise training program significantly changed irisin levels in patients after hip or knee replacement (Table 2). Irisin levels increased after the rehabilitation (*p* < 0.05) by about 0.06 ± 0.16 µg/mL (95%CI: 0.01–0.12) (Figure 1).

**Figure 2:**
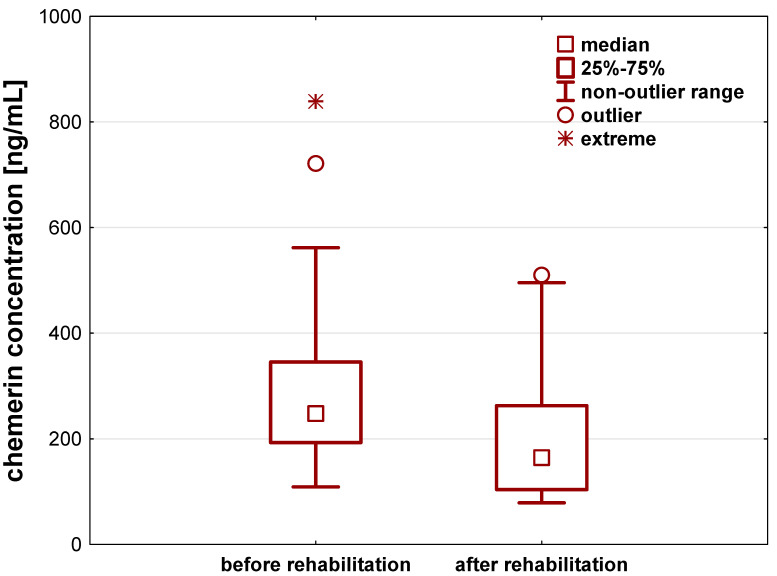
Chemerin concentration [ng/mL] in the serum of patients after hip or knee replacement surgery enrolled to a 21-day individual rehabilitation exercise training program.

Chemerin levels also significantly differed before and after a 21-day individual rehabilitation exercise training program (*p* < 0.001). The rehabilitation decreased chemerin concentration by about 60.9 (33.1–128.5) ng/mL (Figure 2, Table 2).

**Figure 3:**
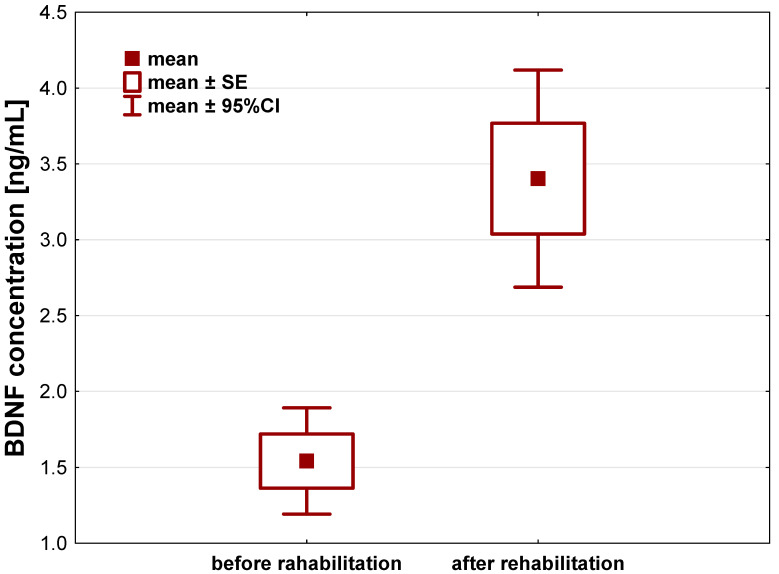
Brain-derived neurotrophic factor (BDNF) concentration [ng/mL] in the serum of patients after hip or knee replacement surgery enrolled to a 21-day individual rehabilitation exercise training program.

In case of BDNF, we observed its statistically significant increase in the patients serum after a 21-day individual rehabilitation exercise training program, compared to its concentration before the rehabilitation started to (*p* < 0.001). In the course of 21 days, BDNF levels increased by about 1.86 ± 1.96 ng/mL (95%CI: 1.22–2.51) (Figure 3, Table 2).

A pairwise comparison of pre-treatment irisin, chemerin, and BDNF serum concentrations results was made, and no correlations were found. A pairwise comparison of post-treatment results for the same factors showed a weak negative correlation for irisin and BDNF (rho = −0.351, *p* < 0.05), and for chemerin and BDNF (rho = −0.416, *p* < 0.01) serum concentrations.

## 4. Discussion

Hip or knee arthroplasty constitutes a significant percentage of orthopedic surgeries. Arthroplasty improves patients’ motor capacity and their quality of life [28,29]. A hip arthroplasty procedure is recognized as one of the most common and most significant operations improving patients’ quality of life [30]. According to OECD data, access to arthroplasty treatment improved by about 7% from 2000 to 2009. Günsche et al. calculated the age-standardized incidence rates for total hip or knee replacements based on OECD data [31]. The authors found that the age-standardized incidence rates for total hip replacement is positively related to incidence and length of stay of coxarthrosis, age-standardized incidence rates for total knee replacement, health expenditures, number of nurses, and social insurance. On the other hand, diabetes prevalence, gross domestic product, and the number of doctor consultations negatively influence the age-standardized incidence rates. In contrast, the total knee replacement rate is positively influenced by health expenditures and the incidence rate of gonarthrosis, and negatively by the number of primary practitioners [31]. In Poland, the number of arthroplasty surgeries increased by 20% in 2017 [32]. Unfortunately, data on osteoarthritis (OA) prevalence, the frequency of its clinical phenotypes, and the physical disabilities it generates, or on the OA’s economic impact on the health system in Poland are non-existent.

In the presented study, we observed that a 21-day individual rehabilitation exercise training program led to a significant increase in irisin and brain-derived neurotrophic factor (BDNF), and a decrease in chemerin serum concentration in patients after hip or knee replacement surgery. Exercise is an effective non-pharmacological intervention that improves physical capacity, body functions, and health. Since the correlation between the beneficial effects of exercise and exercise intensity or duration shows the dose–response relationship, it seems that individually-adjusted rehabilitation exercise training is crucial for patients after hip or knee replacement surgery. In vitro and in vivo studies demonstrated that irisin influences bone cells [33,34,35,36].

Irisin’s effect on bone cells was demonstrated in several in vitro and in vivo studies [27,28,29,30]. Irisin stimulates osteoblasts’ differentiation, their activity, and increases osteocytes’ viability. Simultaneously, irisin affects the osteoclasts in two ways: indirectly, through the increased expression of osteoprotegerin (OPG) in osteoblasts, and directly, as a counter-regulatory hormone increasing osteoclast progenitors differentiation and promoting bone resorption [33,34,36,37]. Colaianni et al. [38] have demonstrated that irisin affects all stages of osteoblast differentiation: the early stage, by increasing the number of ALP+ colonies, and the late stage, by enhancing the mineralized nodules formation [38]. In adults, irisin levels are affected by age, gender, obesity, and muscle mass [39]. Apart from improving the bone strength, irisin has also more broad effects, like increasing energy expenditure and improving cognition [38,40,41]. Anastasilakis et al. [42] demonstrated that the basic irisin level did not depend on the degree of physical activity, but it increased after 20 min of intense muscle exercise [42]. Kurdiova et al. [43] also reported that irisin levels are related to the usual degree of physical activity and to muscular strength, contractility, and volume [43]. According to Anastasilakis et al. [42] and Loffler et al. [39], acute and strenuous exercise increase irisin blood concentration, but they do not change after long-term exercise (6 weeks/1 year).

In the past decade, scientific reports have focused on the responses of irisin to various exercise patterns and types of physical activity. Sprint-type exercises led to an acute increase in the peripheral concentration of irisin in Greyhound dogs [44] and in humans [45,46]. Some studies showed that high-volume resistance exercises engaging all muscle groups led to an increase in the irisin concentration 1 h after exercise [45,47,48], whereas irisin concentration remained unchanged when the exercise engaged only one muscle group [49]. Similarly, the chronic whole-body vibration exercise also increased irisin concentration [50].

However, the meta-analysis [51] of three randomized controlled trials showed that chronic resistance exercise training has a moderate and significant effect on circulating irisin and decreases it compared with the control, and endurance exercise training has only a similar but not significant trend. Similar analysis [51] of nine non-randomized studies revealed that regular exercise training was associated with a small and non-significant overall effect and decreased irisin levels compared with the baseline. On the other hand, Gaudio et al. [52] reported that physical activity positively increased serum irisin levels and bone turnover markers in competitive footballers, when compared to similar subjects with a predominantly sedentary lifestyle [52]. However, in pathophysiological conditions, reduced circulating irisin levels were reported in patients with chronic kidney disease or T2DM, preeclamptic women during gestation, and osteoporotic patients [53].

Here, we report that a 21-day individual rehabilitation exercise training program increased irisin serum concentrations. The assumption of the applied individual rehabilitation exercise training was to ensure that each of the patients, for 21 consecutive days, completed the individually-adjusted exercise sessions that consisted of 30–45 min of aerobic walking, 20–30 min of strength training, and 30–45 min of rotor/bicycle training. The physiotherapy and the living activity training focused on improving the patient’s walking functionality, and engaged all body muscle groups. The obtained results agree with the studies that reported increased levels of irisin after high-volume resistance exercises engaging all muscle groups [45,47,48]. Due to its numerous biological roles, irisin is a prospective therapeutic target, and it offers a new potential foundation for physical therapy [54]. Nevertheless, further studies are needed to determine clinical significance of irisin as the potential marker of successful rehabilitation exercise training of OA patients.

Chemerin presence is related to inflammatory processes such as psoriasis, obesity, metabolic syndrome, hypertension, angina, and cancer [55]. High chemerin concentrations induce MMP-2, MMP-3, MMP-13, and IL8, which accompany joint cartilage degradation [56]. However, studies on chemerin in OA patients are infrequent, and give diverse results. Valcamonica et al. [57] observed high chemerin levels in patients with knee OA. In this study, chemerin levels were related to C-reactive protein, IL-6, and TNF-α levels. The authors suggested that chemerin comprises an inflammatory component [57]. In their previous study, Valcamonica et al. [57] reported contradictory results, showing no significant difference in serum chemerin levels in 11 OA patients, 8 psoriatic arthritis, and 18 rheumatoid arthritis patients. On the contrary, Ma et al. [58] observed higher chemerin levels at the synovial fluid and membrane level in patients with knee OA than in the control group. Similarly, Huang et al. also reported increased chemerin levels in the serum and synovial fluid of patients with knee OA [59]. Bozaoglu et al. [60] related serum chemerin levels to metabolic syndrome components because, in glucose tolerant subjects, plasma chemerin levels were significantly associated with BMI, circulating triglycerides, and blood pressure. We noted a significant decrease in chemerin levels in patients after knee or hip replacement surgery, who underwent the 21-day individual rehabilitation exercise training program. Our findings are similar to the results by Stefanov et al. [61], who reported that a 6-month exercise program combining endurance and resistance exercises led to a statistically significant reduction in serum chemerin concentration in middle-aged, overweight or obese, non-diabetic individuals [61].

Physical exercise (muscle contraction) is an important modulator influencing the production of cytokines such as BDNF [62]. Systematic reviews [59,63] and meta-analyses [64,65] conclude that an acute bout of physical activity transiently increases BDNF peripheral levels. Additionally, chronic physical activity increases BDNF response to an acute bout of physical activity [65]. On the other side of the spectrum, physical inactivity determines the activation of systemic inflammatory pathways in chronic diseases [62]. Low BDNF levels are associated with aging and several diseases: neurologic [65], psychiatric [66], frailty syndrome [67], and impaired cognitive function [68]. Elevated BDNF concentrations that follow intervention (like physical therapy) [69] and physical activity [70] suggest that BDNF may be an important regulatory factor in the elderly population. The results presented here demonstrate increased BDNF serum concentrations after 21 days of regular and moderate exercise. Our previous study showed that the 21-day general alternative rehabilitation exercise training also improved the clinical parameters, such as blood morphology, dyslipidemia, BMI, oxidative stress markers, and the patient’s overall fitness measured with the six-minute walking test (6MWT) of elderly patients after hip or knee replacement surgery due to OA [71]. Since the studies on BDNF plasma concentrations and inflammatory diseases are scarce, it may also be possible that initially low BDNF serum levels are not related to OA, but are instead associated with chronic inflammatory conditions related to aging, as suggested by Gomes et al. [72] and Vasto et al. [73]. Nevertheless, even in light of this possibility, the presented study brings novel data to this discussion.

We are aware of two limitations of the presented study. The first one is related to the number of patients included in the study. It was mostly due to successive loss of patients or withdrawals. For some patients, it was difficult to comply with the rehabilitation program schedule due to personal reasons, or problems with mobility or transport. The second limitation is related to the study design, which does not include a control group consisting of healthy individuals.

## 5. Conclusions

A 21-day individual rehabilitation exercise training program led to a significant increase in irisin and brain-derived neurotrophic factor (BDNF), and a decrease in chemerin serum concentration in patients after hip or knee replacement surgery. Selected markers are widely studied in the context of various diseases, and considered potential new markers of these pathophysiological conditions. Irisin and BDNF seem best choice for that use, however, more research in this area is necessary.

## Figures and Tables

**Table 1:** The inclusion and exclusion criteria for osteoarthritis (OA) patients after hip or knee replacement surgery enrolled to a 21-day individual rehabilitation exercise training.

Inclusion Criteria	Exclusion Criteria
≥18 years old	inflammatory disorders
osteoarthritis	active infection
hip or knee replacement surgery in the past 90 days	active coronary artery disease
	heart failure
	renal or hepatic insufficiency
	diabetes
	hormonal replacement therapy
	antioxidants supplementation in the last 3 months

**Table 2:** Irisin, chemerin, and brain-derived neurotrophic factor (BDNF) concentrations in the serum of patients after hip or knee replacement surgery, before and after a 21-day individual rehabilitation exercise training program.

Analyzed Factor	Concentration before Rehabilitation	Concentration after Rehabilitation	t	*p*
Irisin (µg/mL)	0.43 ± 0.15	0.49 ± 0.13	2.41	<0.05
Chemerin (ng/mL) *	247.5 (193.0–345.3)	164.1 (103.9–264.4)	7.20	<0.001
BDNF (ng/mL)	1.54 ± 1.1	3.4 ± 2.25	5.84	<0.001

Legend: * log-transformed data, BDNF—brain-derived neurotrophic factor.

## Data Availability

The data presented in this study are available on request from the corresponding author.
